# A Skulking Accuser

**Published:** 1890-04-19

**Authors:** 


### The Hospital

A Weekly Journal of

Science, Medicine, Nursing, and Philanthropy.

*For Hospitals, Asylums, Medical Practitioners, Students, Nurses, Families, Parishes, Congregations, and the Charitable Public.*

Vol. VIII.—No. 186.] [April 19, 1890.

### A Skulking Accuser.

Some time ago a pamphlet, by an anonymous author, was sent anonymously to us, which contained charges of discreditable mismanagement against the Committee of the Sussex County Hospital at Brighton. It is not a good rule to spend energy on anonymous publications; but the charges against the Brighton committee were so serious, and the writer of the pamphlet seemed so very sure of his ground that we thought it imperative to depart from our usual custom. Accordingly the pamphlet was considered on its merits; its more important charges were set forth in order; and certain obvious errors were pointed out. Our hope was that the anonymous author would put off his mask; stand forth in his own proper person, and make his charges openly to the Hospital Board. We assumed that if this were done the Committee would either disprove or admit the truth of the accusations; and that the result would be either a reformation or a vindication of the management. Such a line of conduct would have been English and right, and would have commended itself to the approval of every fair-minded man.

But what was the course actually taken by this self-appointed champion of good morals? He proceeds forthwith to republish his pamphlet, and to add thereunto. One of his additions is the article which appeared in this journal, and it is cribbed bodily and published *in extenso* without one single word of acknowledgment of any kind. So far as appears it might have been an original supplement written by the author of the pamphlet himself. But a worse breach of good manners even than this immediately succeeds the unauthorised "conveyance" of The Hospital's article. The new part of the pamphlet is brought to a close by a "letter" which purports to have been addressed to the editor of The Hospital. It is, however, a fact which admits of no contradiction that the editor of The Hospital never received any such letter. Presumably, therefore, no such letter was ever sent to him, or, indeed, was ever intended to be sent. What epithet is it proper to apply to conduct of the kind displayed by this anonymous pamphleteer? It is certainly cowardly, and it is as assuredly mean. There is seldom any excuse for shooting from behind a hedge; in this case there is not a shadow of apology.

It will not surprise anybody to find that the managers of the Brighton Hospital are able to furnish a complete reply to the attacks of which they have been made the objects. We publish on p. 41 a letter from Lieutenant-General Bourchier, secretary to the Hospital, written by order of the Committee of Management. That letter, as will be seen, takes up the various charges in detail, and proves beyond doubt that where they are in any sense true they are grossly exaggerated, and where they are not exaggerated they are not true. The head and front of the Committee's offending was said to be its extravagance. In our former article, we pointed out that this charge was not sustained by facts, and by comparison with the expenditure of other hospitals. The writer of the pamphlet, however, in the belated letter which he says he addressed to The Hospital, but which the Editor of The Hospital has seen for the first time at the tail end of his second pamphlet, returns to the charge of extravagance. This charge, the possession of abundance of material for comparison, enables us to deal with, and not only us, but any person who will take the trouble to consult the pages of the *Hospital Annual*. At p. 13 of the *Annual* a table is given, which shows, among other things, the cost per bed per annum of about fifty country hospitals more or less resembling that at Brighton. The average cost per bed per annum of all these hospitals is £57 0s. 8d.; the cost per bed at Brighton is £71 14s. 8d., and is thus in excess of the average cost by about £14 per bed per annum. But whilst this is so there are several country Hospitals that closely approach in cost per bed that at Brighton, and some, as we pointed out in a previous article, that decidedly exceed the Brighton expenditure.

But we have been reminded that Brighton is "London by the seaside"; and on this ground it is said that the expenditure at Brighton ought not to be compared with the expenditure of provincial hospitals, but with that of those general hospitals in London which have no medical schools. All sorts of provisions are said to be quite as costly at Brighton as in the metropolis, more costly, indeed, than they are in the East and North and South of London. If this view be accepted, the Brighton Hospital will be found to have an expenditure as much below the metropolitan average, as above the provincial. The average cost per bed per annum at the twelve general hospitals in London, which have no medical schools, is £86 13s. 0¼d., or about £14 per bed in excess of Brighton. This comparison throws at least a new light on the question; and most people who know anything about Brighton, will be inclined to agree with the suggestion that it is at any rate as fair to compare the cost of living at Brighton with that of living in London, as to put Brighton in the category of ordinary provincial towns. Whether that be so or not, we think that even the pamphleteer himself will be inclined to agree that Brighton may be placed midway between London and the provinces in point of costliness of living. If this be allowed, and no fair-minded man will question its justice, then, it is seen at once that the cost per bed per annum is almost as exactly as it can be on a level with the average of all the similar hospitals in London and the country.

There are two additional points which, in fairness to the Brighton Committee, ought to be mentioned. The managers believe in feeding their patients well; and so do we. A comparison of the cost of provisions at Brighton with the cost at other similar hospitals, shows that the Brighton Committee spends £7 per bed per annum more on daily necessaries than the general average. This, we think, is entirely to their credit. Many hospitals in the provinces practice, not economy, but parsimony, to the great injury of their patients. No doubt the average cost per bed is thus reduced, but the saving is not a merit but a crime. The other point to be mentioned is that there are no separate accounts kept for the Out-patient Department, or for the Convalescent Home which is associated with the hospital. The expenditure under all these various departments is included under one general heading. But the Hospital treats no less than six thousand four hundred and fifty-nine out-patients, at a cost of probably not less than £500. If this amount, together with the expenses of conducting the Convalescent Home, be deducted from the total expenditure of the Hospital, it will still further reduce the cost per occupied bed by probably a seventh.

What then may be considered to be the net result of the strictures of this literary Dick Turpin who shoots from behind hedges with a mask on his face? A certain amount of discredit, not to say disgrace, has emerged. But the discredit and the disgrace do not attach to the Committee, but to their assailant. This is usually the result of anonymous scribbling. The man who has a good cause in hand does not sneak about in byeways and cellars, but comes out into the market place, and proclaims his message from the house tops. What does this man do when he is offered an opportunity of meeting the Committee, and substantiating his charges by evidence? He declines the challenge. Nothing more needs to be be said. If he were a dog it would be proper to tie a tin kettle to his tail and to send him howling and discredited through the streets. Being, however, as we presume, a man, he must be left to bear, as best he can, the odium he has incurred by his ill-considered and unjustifiable deeds.

